# Hydrogel-Based Treatment of Diabetic Wounds: From Smart Responsive to Smart Monitoring

**DOI:** 10.3390/gels11080647

**Published:** 2025-08-15

**Authors:** Xinghan He, Yongyi Wei, Ke Xu

**Affiliations:** 1 Zhejiang Provincial Engineering Research Center of New Technologies and Applications for Targeted Therapy of Major Diseases, College of Life Science and Medicine, Zhejiang Sci-Tech University, Hangzhou 310018, China; 2022332871021@mails.zstu.edu.cn (X.H.); 2022332864086@mails.zstu.edu.cn (Y.W.); 2 Key Laboratory of Advanced Textile Materials & Manufacturing Technology, Ministry of Education, Zhejiang Sci-Tech University, Hangzhou 310018, China

**Keywords:** hydrogels, diabetic wound, smart responsive, smart monitoring

## Abstract

Diabetic wounds are characterized by a refractory healing cycle resulting from the synergistic effects of hyperglycemic microenvironment, oxidative stress, bacterial infection, and impaired angiogenesis. Conventional hydrogel dressings, with limited functionality, struggle to address the complexities of chronic diabetic ulcers. Smart hydrogels, possessing biocompatibility, porous architectures mimicking extracellular matrix, and environmental responsiveness, have emerged as promising biomaterials for diabetic wound management. This review systematically elucidates the specific response mechanisms of smart hydrogels to wound microenvironmental stimuli, including pH, matrix metalloproteinase-9 (MMP-9), reactive oxygen species (ROS), and glucose levels, enabling on-demand release of antimicrobial agents and growth factors through dynamic bond modulation or structural transformations. Subsequently, the review highlights recent advances in novel hydrogel-based sensors fabricated via optical (photonic crystal, fluorescence) and electrochemical principles for real-time monitoring of glucose levels and wound pH. Finally, critical challenges in material development and scalable manufacturing of multifunctional hydrogel components are discussed, alongside prospects for precision diagnostics and therapeutics in diabetic wound care.

## 1. Introduction

Diabetes mellitus is a metabolic disorder characterized by chronic hyperglycemia, frequently complicated by diabetic foot ulcers (DFUs) and other comorbidities. The cutaneous wounds resulting from these foot ulcers often exhibit delayed or non-healing characteristics [1,2], which significantly impair patients’ quality of life and physical and mental well-being, while imposing substantial economic burdens on healthcare systems globally. In 2021, the global diabetic population reached 529 million, with projections indicating a surge to 1.31 billion by 2050. Notably, over 25% of diabetic individuals suffer from these chronic wounds, leading to clinical manifestations including persistent pain, fever, bacterial infection, limb amputation, and even mortality [3,4].

Diabetic wounds, as a severe health complication, have emerged as one of the critical challenges confronting global healthcare systems. The healing process of these wounds is impeded by multiple factors, often leading to the formation of specialized wound microenvironments characterized by hyperglycemia, chronic inflammation, impaired angiogenesis, and bacterial infections. Notably, the hyperglycemic milieu within wounds predisposes to microbial colonization. Sustained hyperglycemia and the accumulation of advanced glycation end-products (AGEs) exacerbate inflammatory responses, triggering excessive production of reactive oxygen species (ROS) by immune cells such as neutrophils. This oxidative stress cascades results in cellular and tissue damage [5,6,7]. Concurrent bacterial infections and oxidative stress further aggravate vascular complications in diabetic patients, manifesting as compromised blood supply, impaired neovascularization, and ischemic-hypoxic conditions in wound tissues. These pathological alterations disrupt enzymatic activities, alter pH homeostasis, and hinder cellular proliferation, extracellular matrix (ECM) synthesis, and intercellular junction formation. The complex interplay of these microenvironmental factors contributes to the refractory nature of chronic diabetic wounds, with tissue necrosis further exacerbating irregular wound formation [8].

Conventional wound dressings (e.g., gauze or bandages) exhibit limited functionality as passive materials, failing to address the dynamic requirements of diabetic wound microenvironments and potentially causing secondary trauma through adherence to wound beds [9]. Hydrogel dressings demonstrate significant therapeutic potential in diabetic wound management due to their excellent biocompatibility, ECM-mimicking three-dimensional porous architecture, exudate absorption capacity, and ability to maintain optimal moist wound environments [10]. The wound healing process can be divided into multiple distinct phases, each characterized by unique physiological and pathological features as well as specific microenvironmental profiles. For instance, the hyperglycemic environment in diabetic wounds impairs cellular proliferation, ECM synthesis, and angiogenesis, while compromising cell migration capabilities. The porous architecture of hydrogels not only facilitates gas exchange and nutrient delivery but also establishes a favorable microenvironment for adhesion, proliferation, and migration of multiple cell types including fibroblasts, keratinocytes, mesenchymal cells, and epithelial cells [11]. However, most conventional hydrogel dressings currently lack the capability to dynamically adapt to the specific microenvironmental demands of wound sites. Hydrogels possessing environmental responsiveness and multifunctionality represent a class of emerging smart dressings that hold significant potential as advanced therapeutic delivery systems.

Numerous previous reviews have summarized the therapeutic advancements of smart hydrogels for diverse types of wounds [12]. Among these, diabetic wounds, characterized by complex pathophysiological mechanisms and chronic non-healing features, have remained a challenging research focus. However, few existing studies have systematically evaluated and reviewed the correlation between hydrogel functionalities and their applications in diabetic wound healing. Meanwhile, conventional reviews primarily emphasize the stimuli-responsive properties of hydrogels while neglecting the core requirements of diabetic wounds. As a representative chronic wound type, diabetic ulcers necessitate not only conventional smart-responsive therapies but also integrated wound monitoring or diagnostic capabilities within hydrogel materials or therapeutic systems. This dual-functional approach aims to facilitate progressive wound repair over extended periods through real-time condition assessment and adaptive treatment modulation [13]. In recent years, hydrogels equipped with therapeutic and wound-monitoring functionalities have emerged as a focal research area in smart dressing development due to their capacity to assess wound microenvironments and deliver on-demand therapeutic interventions. Given this context, this article focuses on diabetic wound management, summarizing advancements in smart-responsive hydrogels for diabetic wound treatment over the past five years. The review systematically addresses key aspects including wound characteristics, gel response mechanisms, and dynamic intelligent monitoring. It analyzes the responsive mechanisms of various smart hydrogels under different microenvironmental conditions, discusses multimodal monitoring approaches applicable during wound repair processes, and aims to provide novel references and insights for the integration strategies of next-generation hydrogel-based biosensors. This work offers theoretical foundations and technical guidance for developing intelligent wound management systems with adaptive therapeutic capabilities.

## 2. Pathophysiological Characteristics of Diabetic Wounds

The pathophysiological characteristics of diabetic wounds are highly complex, with multiple interrelated factors involved. In diabetic wound environments, internal microenvironmental stimuli include pH [14,15], enzymes [16], glucose [17], ROS [18], and matrix metalloproteinase-9 (MMP-9) [8]. Additionally, external macro-environmental stimuli such as temperature [19], light [20], mechanical forces [21], magnetic fields [22], and electric fields [23,24] have been demonstrated to trigger hydrogel transformation, facilitate drug release, stimulate cell proliferation and differentiation, as well as enable molecular signal detection [25].

### 2.1. Effects of Hyperglycemia

One of the most prominent characteristics of wounds in diabetic patients is the hyperglycemic environment, which constitutes a major impediment to healing. Clinically, elevated glucose levels at injury sites disrupt normal cellular metabolic activities, leading to cellular and vascular damage. Concurrently, excessive glucose provides abundant nutrients that create a conducive environment for bacterial proliferation, thereby facilitating recurrent bacterial infections. Hyperglycemia also induces membrane stiffening and vasoconstriction, further reducing blood flow and impairing nutrient and oxygen delivery to the wound site, which compromises tissue repair. Another critical mechanism by which hyperglycemia hinders wound healing involves diminished activity of antioxidant enzymes such as glutathione peroxidase (GSH-Px) and superoxide dismutase (SOD), resulting in oxidative stress-mediated cellular damage [26]. Therefore, in clinical management of diabetic wounds, glycemic control remains a pivotal therapeutic strategy.

### 2.2. Abnormal Oxidative Stress and Inflammatory Response

Clinical studies have demonstrated that oxygen plays a pivotal role in wound healing by modulating angiogenesis, promoting cell proliferation and migration, and interacting with various cytokines. In diabetic wounds, capillary damage and impaired angiogenesis lead to insufficient oxygen supply and compromised immune responses, exacerbating localized inflammation [27]. Notably, AGEs can directly activate immune cells to produce elevated levels of ROS, ultimately inducing oxidative stress, disrupting cellular redox homeostasis, and aggravating metabolic dysregulation in the wound microenvironment. Furthermore, AGEs impair the phenotypic transition of macrophages from classically activated (M1) macrophages with pro-inflammatory properties to alternatively activated (M2) macrophages associated with anti-inflammatory and tissue repair functions. Excessive infiltration of M1 macrophages results in sustained accumulation and activation of pro-inflammatory cells at the lesion site, prolonging the inflammatory phase [28]. When wounds become infected, bacteria may trigger persistent inflammatory responses at the infection site, further impeding the resolution of inflammation and delaying wound healing.

### 2.3. Bacterial Infection

Usually, the pH value of the skin surface is typically mildly acidic. Upon wound formation, the underlying subcutaneous tissue with its inherently mildly alkaline environment becomes exposed. During normal wound healing, the pH transition generally shifts from alkaline to acidic; however, in chronic and infected wounds, prolonged inflammation sustains an alkaline pH environment. This alkaline wound milieu creates conditions more conducive to bacterial proliferation, thereby elevating infection risks. Clinically, the majority of chronic and infected wounds maintain alkaline pH levels [28]. Furthermore, capillary damage combined with impaired angiogenesis leads to insufficient oxygen supply and diminished immune responses, which collectively exacerbate bacterial infection susceptibility.

### 2.4. Angiogenesis Disorders

Under normal physiological conditions, ROS effectively promote angiogenesis and confer resistance against bacterial infections. In diabetic wounds, oxidative stress-induced inflammation leads to collagen and ECM degradation, as well as impaired angiogenesis. Hyperglycemia compromises normal blood perfusion to the wound site by altering hemodynamic properties, resulting in cellular nutrient deficiency. This pathological cascade suppresses angiogenic processes and delays wound healing in diabetic conditions [29]. The homeostatic balance between pro-angiogenic and anti-angiogenic cytokines is disrupted in diabetic patients, with a relative upregulation of inhibitory cytokines that negatively regulate neovascularization. Chronic hyperglycemic states further damage vascular endothelial cells, impairing their migratory capacity toward injured tissues and diminishing their ability to establish new vascular networks. This endothelial dysfunction consequently restricts oxygen and nutrient delivery to wound sites, thereby compromising the metabolic support required for effective angiogenesis.

**Figure 1:**
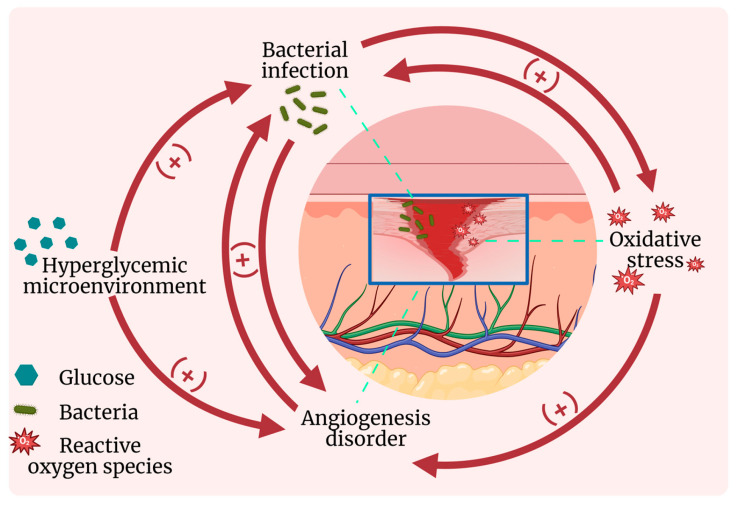
Interaction diagram of physiological and pathological characteristics of diabetic wound.

In general, hyperglycemia directly reduces antioxidant enzyme activity, provides nutrients for bacterial proliferation, and impairs angiogenesis. Meanwhile, aberrant oxidative stress and inflammatory responses exacerbate vascular damage and collagen degradation while compromising immune defenses, thereby promoting bacterial infections and further suppressing angiogenesis. Bacteria thrive more readily in the nutrient-rich environment provided by hyperglycemia and the alkaline conditions resulting from inflammation, with persistent inflammation subsequently intensifying oxidative stress and angiogenic dysfunction [29]. Angiogenic impairment leads to insufficient oxygen and nutrient supply, which in turn aggravates oxidative stress, inflammatory responses, and bacterial infections. These four key factors—hyperglycemia, oxidative stress, inflammation, and bacterial infection—are interconnected and causally linked, forming a self-perpetuating vicious cycle that collectively hinders diabetic wound healing (as illustrated in Figure 1).

## 3. Smart Response of Hydrogels to Wound Microenvironment Modulation

Diabetic wounds present a complex pathophysiological environment where multiple factors impede the normal healing process [30]. Traditional wound dressings, such as gauze bandages, can achieve hemostasis, absorb wound exudate, and provide a protective barrier against infection. However, these materials fail to accelerate wound healing. Frequent dressing changes may even cause secondary or multiple injuries, compromising patient compliance. According to the modern moist wound healing theory, ideal dressings should maintain an optimal microenvironment with appropriate humidity, temperature, and pH value, while being removable without damaging skin tissues. Furthermore, dressings with integrated hemostatic properties, anti-infective capabilities, and pro-regenerative functions are particularly desirable for diabetic wounds. Currently available moist wound dressings mainly include foams, films, and hydrogels. Among these, hydrogels demonstrate superior advantages in biocompatibility, moisture retention, and optical transparency, enabling both therapeutic intervention and visual monitoring of wound progression [31,32]. To address clinical limitations of conventional diabetic wound dressings, recently developed smart-responsive hydrogels not only exhibit excellent moisturizing properties, biocompatibility, drug loading capacity, and controlled release mechanisms [30,33], but more importantly, they can sense and respond to dynamic changes in the wound microenvironment (e.g., pH, enzymes, ROS, and glucose concentration; see Table 1). This enables on-demand release of bioactive agents, inflammation modulation, ROS scavenging, and real-time wound status monitoring through biosensing functions [34]. The dynamic interaction between responsive hydrogels and wound microenvironment allows precise therapeutic interventions, marking a significant advancement in diabetic wound management [35].

The design strategies for smart-responsive hydrogels involve leveraging specific biomarkers within the wound microenvironment as triggering stimuli to induce physical or chemical transformations in the hydrogel, thereby enabling the execution of predefined therapeutic functions. Based on distinct triggering signals, the responsive mechanisms can be categorized into the major types outlined below.

### 3.1. pH Response

**Figure 2:**
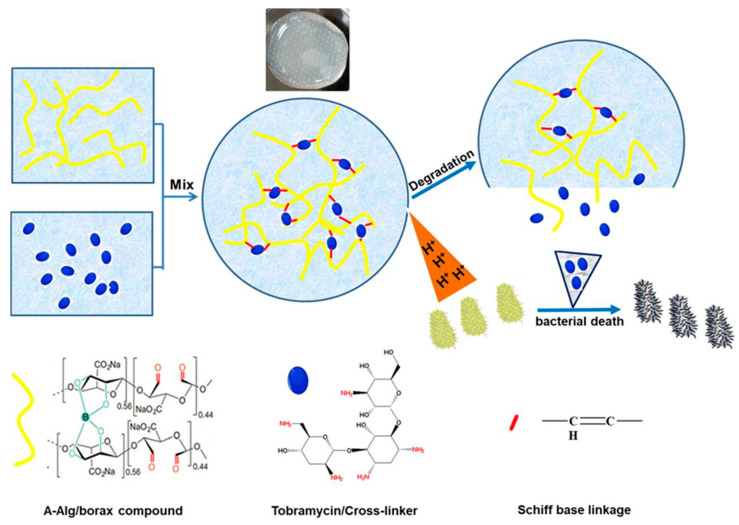
Degradation and drug release behavior of pH-responsive hydrogels under acidic conditions. Reproduced with permission from [50], Colloids Surf B Biointerfaces, 2022.

A prominent pathological characteristic of diabetic wounds in the inflammatory phase is the aberrant acidic microenvironment, typically ranging from pH 4.5 to 6.5 [48]. This acidic deviation from physiological pH (~7.4) provides a critical trigger signal for designing pH-responsive intelligent hydrogels. Dynamic covalent bonds (e.g., Schiff base linkages -C=N- and borate ester bonds -B-O-) with acid-labile properties serve as crosslinking junctions or “gatekeeper switches” in hydrogel networks. Acidic conditions specifically induce hydrolysis or weaken the bond strength, leading to hydrogel network dissociation, increased porosity, accelerated degradation, or controlled drug release. This chemically bond-cleavage-based responsiveness mechanism demonstrates superior rapidness and precision. For Schiff base bonds, protonation of imine groups (-C=N-) under acidic conditions significantly reduces bond stability, facilitating hydrolytic cleavage. For instance, Schiff base-based hydrogels can utilize low pH to trigger on-demand release of antibacterial agents in infected wounds, effectively inactivating bacteria [49]. As illustrated in Figure 2, our previous study [50] demonstrated that tobramycin (represented as blue small ellipses), an aminoglycoside antibiotic, can form dynamic Schiff base bonds (red dots) between its amino groups and the aldehyde groups (yellow lines) in oxidized sodium alginate through reversible chemical interactions, thereby enabling gelation of the precursor solution. In infected diabetic wounds, bacterial metabolic proliferation degrades surrounding organic matter into lactic acid and carbonic acid, establishing an acidic microenvironment. The H+ ions generated in this environment can disrupt the previously formed crosslinking points within the hydrogel, leading to the cleavage of the Schiff base bonds. Consequently, tobramycin is released, effectively killing the bacteria in the environment, while simultaneously promoting the degradation of the hydrogel network. This process establishes a dynamic balance between the drug release and the bacterial load, thus preventing the misuse of antibiotics. Additionally, Sun et al. constructed an ODex/BSA-Zn hydrogel through pH-sensitive Schiff base bonds formed between aldehyde groups of oxidized dextran (ODex) and amino groups of bovine serum albumin (BSA), combined with Zn^2+^-BSA coordination interactions to create a dynamic network with pH-responsive properties [39]. Regarding borate ester bonds, acidic environments promote their hydrolysis or reduce binding affinity. For example, borate ester-based hydrogels exhibit accelerated release of loaded nanoparticles under acidic conditions through disruption of crosslinking networks [51]. Notably, advanced pH-responsive hydrogels not only passively adapt to wound acidity but also actively modulate local pH to intervene in pathological processes. Zhou et al.’s DFO/ENZ@CPP hydrogel actively regulates wound pH, decreasing it from initial physiological pH 7.4 to 5.8 on day 3. This artificially induced moderate acidic microenvironment promotes macrophage polarization toward reparative M2 phenotypes, thereby modulating immune responses to facilitate healing [52], demonstrating the advanced functionality of pH-responsive hydrogels transitioning from passive sensing to active intervention.

### 3.2. Enzyme Response

**Figure 3:**
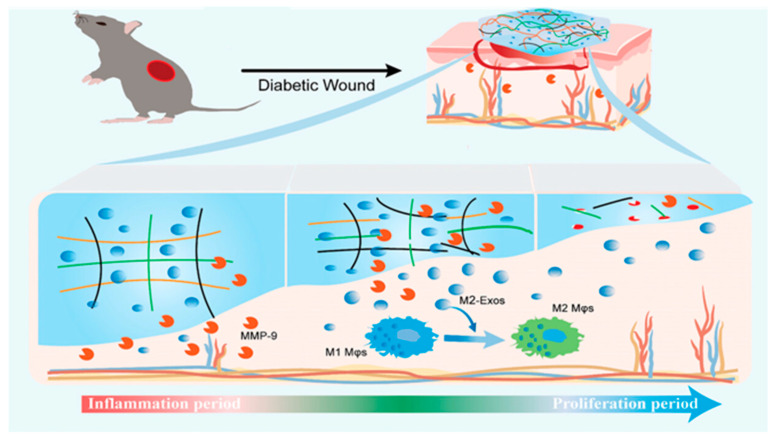
A smart MMP-9-responsive hydrogel that enables on-demand M2-Exo release to promote timely M1–M2 Mφ switching and accelerate diabetic wound healing. Reproduced with permission from [40], Advanced Healthcare Materials, 2025.

Matrix metalloproteinases (MMPs) are enzymes capable of degrading most ECM proteins, which facilitate cell migration by dismantling ECM and basement membrane barriers, thereby contributing to tissue regeneration and wound healing [53,54,55]. In cases of impaired chronic wound healing, such as diabetic ulcers, prolonged inflammatory responses induce aberrant overexpression of MMPs, leading to delayed tissue regeneration and wound closure failure. This pathological condition is primarily characterized by excessive activation of MMP-2 and MMP-9, which disrupts ECM homeostasis and impedes the normal wound healing cascade [56,57,58]. To address this key therapeutic target, enzyme-responsive smart hydrogels have been developed as a strategic solution. The core design principle of these hydrogels involves incorporating molecular structures (e.g., specific peptide sequences) sensitive to target enzymes (such as MMP-9) as crosslinking units or “gated” switches. As demonstrated by Meng et al., the Exo@MRH hydrogel network undergoes structural disintegration when MMP-9 concentrations elevate in the wound microenvironment. This mechanism involves MMP-9-mediated cleavage of the PG-6 peptide segment within the hydrogel matrix, which triggers on-demand release of encapsulated therapeutic agents [40]. This targeted drug delivery or degradation behavior not only enables direct delivery of bioactive substances but also modulates abnormal enzymatic activity (e.g., downregulating MMP-9 expression or activity) to improve cellular behaviors, ultimately promoting wound repair. For instance, a smart hydrogel constructed through MMP-9-sensitive peptide crosslinking between oxidized dextran and carboxymethyl chitosan specifically responds to elevated MMP-9 concentrations at inflammatory sites. This responsive system facilitates controlled release of M2 macrophage-derived exosomes (M2-Exos), effectively promoting macrophage polarization from the pro-inflammatory M1 phenotype to the reparative M2 phenotype, thereby significantly accelerating diabetic wound healing (Figure 3) [40].

### 3.3. ROS Response

**Figure 4:**
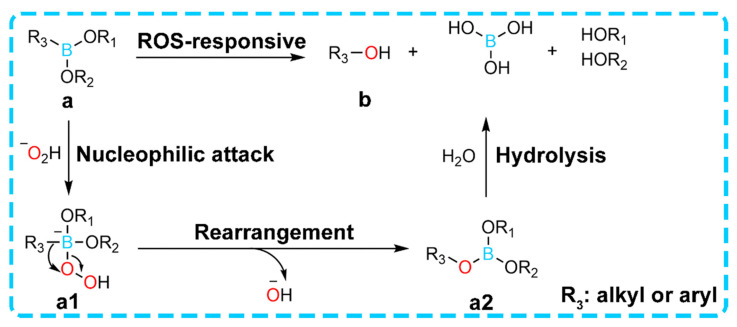
The response mechanism of phenylboronate to ROS. The unoccupied 2p orbital in the boron atom of compound a provides conditions for H_2_O_2_ to act as a nucleophile, on the basis of which the lone pair of electrons on the oxygen atom in the −O_2_H group initiates a nucleophilic attack to form intermediate a1, followed by a rearrangement process similar to the Baeyer-Villiger reaction that generates intermediate product a2, which then undergoes rapid hydrolysis to eventually form product b. This rearrangement step acts as the rate-limiting step of the entire reaction. Both aryl boronic acid esters (e.g., phenylboronate) and alkyl boric acid esters owe their ROS responsiveness to such a rearrangement between the boronic acid group and the oxygen atom of the peroxy bond. Reproduced with permission from [62], Materials Horizons, 2024.

In diabetic wounds, persistent oxidative stress leads to abnormally elevated levels of ROS [59,60,61], which not only directly damages tissues but also delays the repair process during the inflammatory phase of wound healing. To address this pathological feature, ROS-responsive hydrogels have been designed to incorporate ROS-sensitive chemical bonds such as borate ester bonds. Due to their sensitive response to ROS and rapid gelation properties, numerous phenylboronate-based hydrogels have been developed. These hydrogels can be broadly classified into two categories: (1) hydrogels constructed using phenylboronate as a crosslinker, which degrade during the reaction; and (2) hydrogels with phenylboronate-coupled drugs, enabling controlled drug release upon reaction. Notably, when exposed to the high ROS microenvironment of wounds, the chemical bonds break, triggering either hydrogel degradation or on-demand release of encapsulated therapeutic agents [62] (Figure 4).

#### 3.3.1. Responsive Degradation and Drug Release

Wu et al. [41] developed a glucose- and ROS-dual-responsive hydrogel system (HAP-PVA/Reg3α), synthesized from phenylboronic acid-modified hyaluronic acid (HAP) and polyvinyl alcohol (PVA), which exhibited near-complete degradation behavior under simulated diabetic wound microenvironment conditions (high glucose, high H_2_O_2_), demonstrating its high responsiveness to elevated ROS levels. Concurrently, the Reg3α release from this hydrogel showed a significant increase with rising H_2_O_2_ concentrations. In related studies, ROS-responsive PVA hydrogels have been applied for co-delivery of metformin and fibroblast growth factor-21 (FGF-21), leveraging ROS-triggered release mechanisms to synergistically enhance wound healing [42].

#### 3.3.2. Efficient ROS Scavenging Activity

Zhang et al. [51] developed a novel conductive hydrogel (HEPP) composed of hyaluronic acid (HA), caffeic acid-conjugated ε-polylysine (EPL), phenylboronic acid (PBA), and Pt-Pd nanoparticles. This hydrogel integrates a self-cascade ROS scavenging function, effectively regulating high-level ROS to combat infections and modulate inflammation, thereby promoting wound healing in diabetic patients. It is noteworthy that most responsive hydrogels employ metal nanoparticles to enhance ROS scavenging capabilities, where these nanoparticles generally demonstrate favorable biocompatibility at low concentrations but may induce cytotoxicity at high concentrations. Therefore, developing hydrogels with intrinsic capacity to eliminate excess ROS becomes particularly critical [61]. For instance, utilizing materials containing polyphenolic structures (e.g., tannic acid) as gel matrices or incorporating components with antioxidant enzyme-like activities (such as nanozymes) during preparation processes can effectively reduce cytotoxicity.

### 3.4. Glucose Response

**Figure 5:**
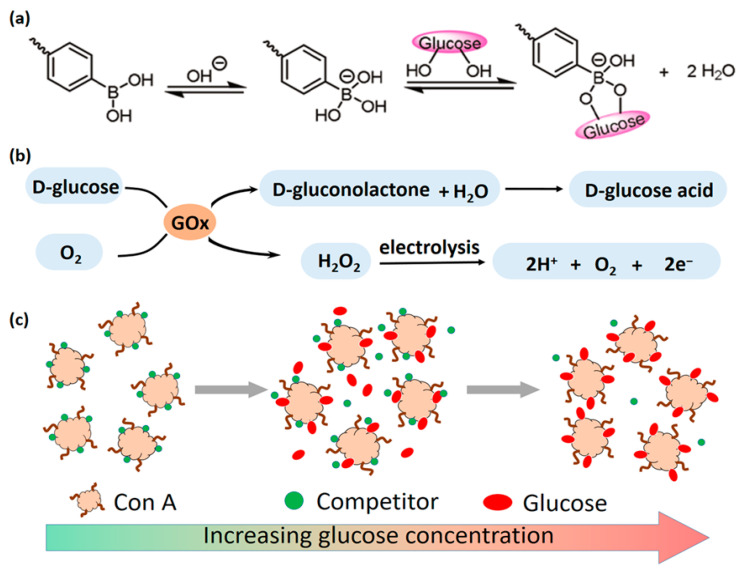
(**a**) Reversible and dynamic binding of glucose to the anionic tetrahedral structure of PBA; (**b**) glucose is enzymatically converted to H_2_O_2_ and D-glucose acid, and H_2_O_2_ is electrolyzed to release electrons; (**c**) Con A has various binding sites for sugar. When free glucose molecules appear around Con A, they will compete for the binding sites with competitors. Through labeling competitors with a fluorophore, the glucose level can be examined. Reproduced with permission from [63] npj Flexible Electronics, 2021.

Hyperglycemia represents a core pathological feature of the wound microenvironment in diabetic conditions. Glucose-responsive hydrogels have demonstrated significant application potential in this field due to their ability to specifically sense and quantify localized glucose concentrations in wound areas. Currently, the primary mechanisms enabling glucose responsiveness include the following: (1) the glucose oxidase (GOx)-catalyzed reaction system, (2) the Concanavalin A (ConA)-mediated competitive binding system, and (3) the phenylboronic acid (PBA)-based reversible binding system, with specific mechanism pathways illustrated in Figure 5.

#### 3.4.1. GOx Catalytic Mechanism

The core mechanism involves integrating GOx into the hydrogel network [64,65,66]. GOx catalyzes the oxidation of glucose, generating gluconic acid and H_2_O_2_. This localized glucose consumption serves as a therapeutic effect by reducing blood glucose levels, while the produced gluconic acid lowers the local pH. The byproduct H_2_O_2_ functions as an ROS. These microenvironmental changes (pH↓, H_2_O_2_↑) can activate responsive components within the hydrogel, such as triggering pH-responsive or ROS-responsive units (e.g., cleavage of sensitive chemical bonds, drug release) [48,60]. Additionally, H_2_O_2_ can participate in Fenton reactions, catalyzed by metal ions (e.g., Fe^2+^) or nanozymes, to generate highly reactive hydroxyl radicals (OH) for antibacterial therapy [67,68]. Furthermore, these reaction products provide sensing signals: gluconic acid-induced pH changes or H_2_O_2_ can act as signal sources, enabling real-time glucose monitoring through coupling with pH-sensitive chromogenic/fluorescent probes or H_2_O_2_ electrochemical/optical detection elements [45,69]. Beyond these effects, the increased ionic strength or H_2_O_2_ production from the GOx reaction can alter hydrogel physical properties (e.g., conductivity). For instance, in a triboelectric nanogenerator (TENG) biosensor, H_2_O_2_ generated by GOx enhances hydrogel conductivity, amplifying signal output for glucose detection [70].

#### 3.4.2. ConA Binding Mechanism

The mechanism is based on the specific and reversible binding capability of Concanavalin A (ConA), a carbohydrate-binding protein, with saccharide molecules. The operational principle relies on competitive binding dynamics: when glucose concentration increases in the environment, free glucose molecules competitively bind to ConA. This competitive interaction alters the intermolecular forces between ConA and immobilized carbohydrate ligands (e.g., carboxymethyl amylopectin) anchored to the hydrogel matrix, inducing dynamic reconfiguration of the hydrogel’s crosslinking network (e.g., dissociation or formation of crosslinking points). These structural changes subsequently trigger hydrogel swelling or controlled drug release behavior [63]. In sensing applications, changes in the binding state of ConA with sugar molecules (such as alterations in hydrogel swelling degree, refractive index, or electrochemical properties) can also be utilized to construct optical or electrochemical glucose biosensors [46]. However, practical applications are limited by the instability of protein molecules and susceptibility to contamination in complex environments, which hinder their clinical translation [71,72].

#### 3.4.3. Competitive Binding Mechanism of Phenylboronic Acid

Phenylboronic acid (PBA) and its derivatives represent the most extensively utilized glucose-responsive components [73,74,75]. The fundamental mechanism relies on the reversible covalent borate ester bond formation between PBA and cis-diol structures (e.g., polyols present in glucose molecules) [47,59,75]. Under hyperglycemic conditions, glucose molecules competitively bind with PBA to form borate ester bonds, which disrupts the pre-existing PBA-diol crosslinking networks, thereby reducing the original crosslinking density [47,76]. This structural disruption triggers hydrogel swelling, degradation, or sol–gel transition [77]. Such conformational changes can be harnessed to modulate the release kinetics of encapsulated therapeutic agents (e.g., insulin [78], metformin [79]), enabling glucose-responsive drug delivery [46]. Additionally, glucose-induced volumetric alterations or impedance variations offer monitoring capabilities through detection of micro-needle height changes caused by hydrogel swelling or signal modifications from embedded chromogenic/fluorescent probes [80].

## 4. Smart Monitoring-Based Integration Strategies for Hydrogel Biosensors

### 4.1. Glucose-Responsive Integrated Smart Monitoring Strategy

In the context of therapeutic management for diabetic wounds, glycemic monitoring serves as a pivotal navigational system that directly guides treatment protocols. By dynamically capturing periwound glucose fluctuations in real time, this approach enables precise modulation of therapeutic strategies, including timely implementation of localized glucose-lowering interventions, optimization of anti-infective regimens, or adjustment of tissue repair-promoting measures. Consequently, the development of patient-comfort-oriented, real-time, and precise glucose monitoring technologies has emerged as a critical research priority. Conventional glucose monitoring methodologies, predominantly invasive in nature, inherently restrict capabilities for continuous surveillance. Recently, wearable and minimally invasive biosensors have garnered significant scientific attention due to their inherent advantages in continuous monitoring, coupled with their capacity to non-invasively or micro-invasively detect physiological fluids such as sweat, tear fluid, or interstitial fluid (ISF) [81,82]. Among these innovative sensing platforms, hydrogels have established themselves as premier candidates for constructing biosensing interfaces capable of detecting specific biomarkers like glucose. This is attributed to their exceptional biocompatibility, mechanical flexibility, and amenability to incorporation of functional components. These hydrogel-based systems transduce biochemical glucose variations into quantifiable optical or electrochemical signals, thereby facilitating real-time or on-demand glucose level monitoring [83].

#### 4.1.1. Optical Sensors

Optical sensors, characterized by their electrode-free nature, robust resistance to electromagnetic interference, and potential for visual detection, demonstrate significant application prospects in the field of glucose monitoring [84,85,86]. By integrating optical sensing mechanisms with hydrogel materials, these systems can achieve highly sensitive and specific detection through volume expansion, optical property alterations, or fluorescent responses triggered by glucose and other analytes. Researchers have systematically explored diverse optical principles synergistically combined with hydrogel matrices, leading to the development of multiple hydrogel-based optical glucose sensor architectures [87,88,89].

##### Photonic Crystal (PC)/Structural Color Sensors

Currently, an intuitive visualization detection approach involves constructing photonic crystal (PC)/structural color sensors. These sensors generate structural colors through periodic nanostructures based on the Bragg diffraction characteristics of photonic crystals [90,91]. The hydrogel matrix typically incorporates glucose-specific binding molecules, such as PBA groups or their derivatives (e.g., 3-acrylamidophenylboronic acid (3-APBA), AFPBA). Upon binding with glucose molecules, these ligands alter the hydrogel’s charge state and hydrophilicity, inducing swelling or deswelling. This volumetric transformation modulates the arrangement or interparticle spacing of photonic crystal structural units (e.g., nanoparticle spacing), thereby shifting the Bragg diffraction wavelength [88]. This wavelength shift manifests as visible structural color changes, enabling glucose concentration visualization [91]. The sensors can be fabricated through nanoparticle self-assembly [92], replica template imprinting, in situ polymerization (e.g., magnetic field-guided nanochain structures) [93], or construction of core–shell architectures [88] and dual-network systems [90]. Furthermore, they can be integrated into microneedle patches for minimally invasive monitoring [83].

**Figure 6:**
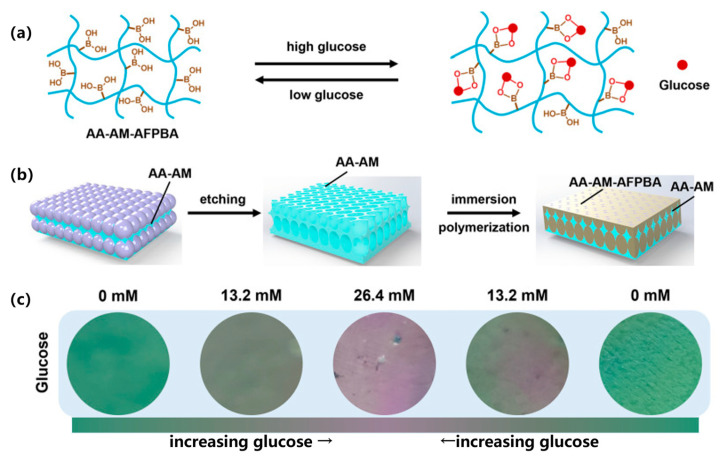
Schematic illustration of the synthesis and glucose response mechanism of photonic crystal hydrogels (PCHs). (**a**) Glucose response mechanism of AA-AM-AFPBA hydrogels. (**b**) Preparation process of double-network PCHs consisted of AA-AM and AA-AM-AFPBA hydrogels. (**c**) Green PCHs reacted with different glucose solutions from 0 to 13.2 mM, 26.4 mM, and back to 13.2 mM and 0 mM. Reproduced with permission from [90], Journal of Nanobiotechnology, 2024.

Recently, Yang et al. [90] developed single-network and double-network photonic crystal hydrogels (PCHs) through the polymerization of acrylamide (AM) and acrylic acid (AA) with glucose-responsive (4-((2-acrylamidoethyl)carbamoyl)-3-fluorophenyl)boronic acid (AFPBA) molecules, combined with the structural color properties of photonic crystal (PC) architectures composed of silica nanoparticles. These hydrogels achieve visual glucose monitoring by exploiting the volume variation induced by glucose–AFPBA interactions, which alters the structural color (Figure 6). The double-network PCHs demonstrated twice the response speed of the single-network system due to their higher volumetric fraction of glucose-responsive motifs. Notably, the glucose levels measured in a murine diabetic wound model using these PCHs showed excellent consistency with tail–vein blood measurements (19.0–19.6 mM).

##### Fluorescence Sensors

**Figure 7:**
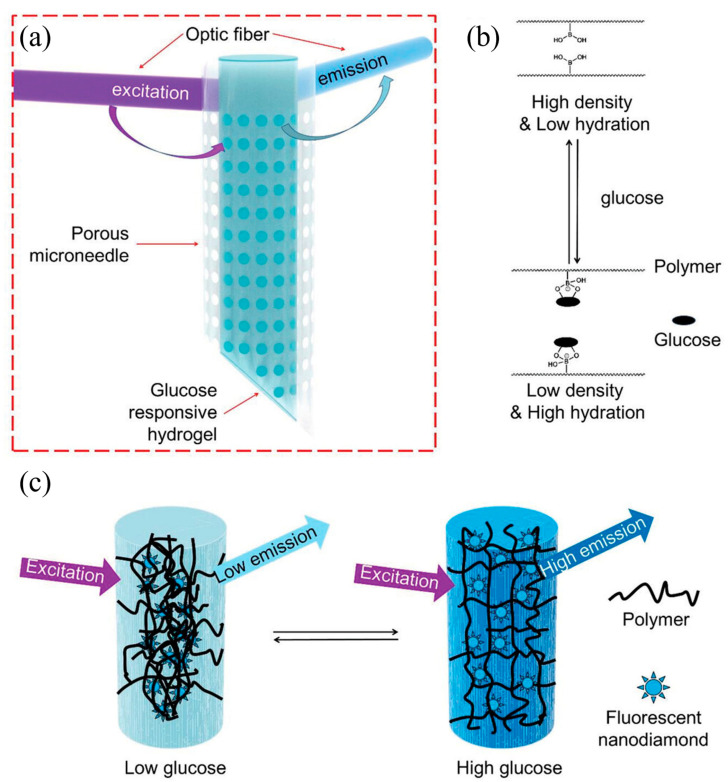
Schematics of the microneedle with fluorescent nanodiamond-based hydrogel. (**a**) Schematic illustration of the microneedle device comprising a transparent porous wall and covalently bound fluorescent nanodiamond-based boronic hydrogel for glucose sensing. (**b**) The reversible complexation of the boronic acid group and cis diols of glucose molecules enables the reversible changes of the polymer network at different glucose concentrations. (**c**) Schematic illustration of the glucose-responsive hydrogel that can change fluorescence output according to glucose concentration. Reproduced with permission from [84], Advanced Science, 2023.

Besides structural color changes, fluorescent sensors represent another crucial optical sensing technology, which utilizes variations in fluorescent signals (e.g., intensity, wavelength, lifetime) to indicate analyte concentrations [86]. In hydrogel-based fluorescent glucose sensors, fluorophores such as nanodiamonds (NDs), quantum dots (QDs), carbon dots (CDs), or organic dyes are typically integrated with glucose-responsive molecules (e.g., phenylboronic acid groups [94], aptamers [83], or enzymes [85,87]) and immobilized within the hydrogel network [84,95]. The binding or reaction with glucose induces microenvironmental changes in fluorophores, conformational alterations, or modulates fluorescence resonance energy transfer (FRET) and photoinduced electron transfer (PET) processes, thereby generating detectable fluorescent signal variations. For instance, in enzyme-based sensors, GOx catalyzes the consumption of molecular oxygen by glucose, where oxygen functions as a quenching agent for specific fluorophores. Consequently, the resulting variations in fluorescence signals—particularly the phosphorescence lifetime—can be quantitatively correlated with glucose concentration [87]. These sensors are fabricated by doping, copolymerizing, or immobilizing fluorophores, sensing molecules, and enzymes within hydrogel matrices, with platforms ranging from optical fibers to microneedle arrays [96]. ND-based fluorescent sensors demonstrate excellent photostability [84]. Zhang et al. [84] developed a fluorescent nanodiamond-boronate hydrogel for continuous glucose monitoring (CGM) through covalent integration of surface-functionalized fluorophores via radical copolymerization. This system employs glucose-boronate reversible binding-induced network structural changes to modulate fluorescence intensity. Combined with porous microneedles (for ISF extraction via capillary action) and optical fiber signal transmission, the device achieved continuous glucose monitoring (Figure 7). It demonstrated high stability (reliable sensitivity after 3 months), excellent biocompatibility (minimal inflammatory response), and accurate monitoring performance (consistent with commercial instruments) in both small and large animal models. In the application aspect, Yang et al. developed a multifunctional integrated hydrogel dressing (PF127@Zn/C-G) for wound glucose monitoring and accelerated healing. The GOx incorporated in the hydrogel catalyzes the oxidation of wound-exudate glucose to generate gluconic acid, leading to their decomposition and subsequent activation of carbon dot (CD) fluorescence for real-time glucose monitoring. This system simultaneously retained the excellent antioxidant capacity and reactive oxygen species (ROS)-scavenging activities of CDs, while the sustained release of Zn^2+^ ions promoted angiogenesis, thereby synergistically facilitating the management of diabetic wounds [97].

##### Noble Metal Nanoparticle Sensors

To achieve higher sensitivity or harness optical phenomena at the micro-/nanoscale, researchers have developed alternative types of hydrogel-based optical sensors. Surface-enhanced Raman scattering (SERS) sensors employ noble metal nanoparticles, such as silver nanoparticles (AgNPs), doped or immobilized within hydrogel matrices to amplify Raman scattering signals from adjacent glucose molecules for detection [95,98]. Hydrogels can be fabricated into microspheres incorporating noble metal nanoparticles as SERS-active substrates [98]. Another class of sensors exploiting the optical properties of noble metal nanoparticles is Localized Surface Plasmon Resonance (LSPR) sensors. These typically integrate noble metal nanoparticles through doping or covalent fixation within glucose-responsive hydrogel matrices (e.g., PBA-functionalized polyacrylamide) [86]. The hydrogel’s glucose-induced swelling alters either the interparticle distances between nanoparticles or the refractive index of the surrounding medium [83], resulting in shifts in LSPR peak positions or changes in absorption/transmission intensity. Such sensors enable qualitative and continuous glucose monitoring with excellent flexibility and stable optical readouts. They can be engineered as composite materials or even integrated onto optical fiber tips [96].

**Figure 8:**
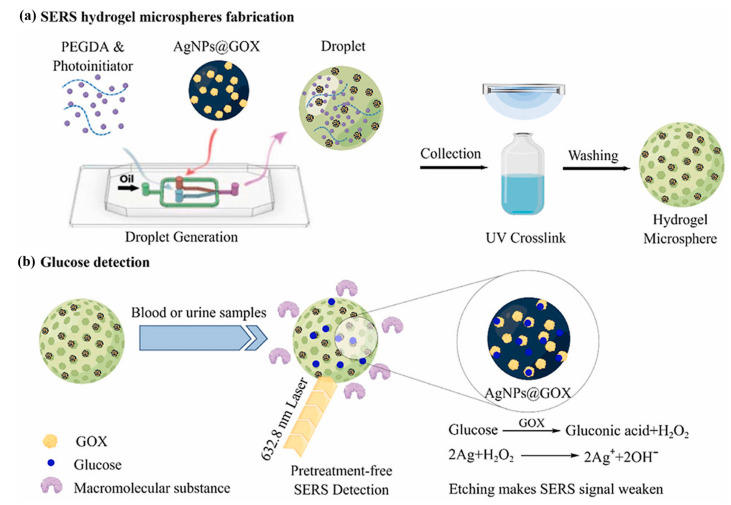
Overview of a workflow of the SERS active substrate based on hydrogel microspheres for pretreatment-free detection of glucose in biological samples. Reproduced with permission from [98], Talanta, 2023.

Wang et al. [98] developed a glucose biosensing platform by utilizing poly (ethylene glycol) diacrylate (PEGDA) as the matrix combined with GOX-modified silver nanoparticles (AgNPs@GOX). Through microfluidic droplet technology, monodisperse hydrogel microspheres with precisely controlled pore sizes were fabricated. The tunable pore architecture selectively permits the diffusion of small-molecule glucose while effectively blocking large molecular interferents, thereby enabling label-free detection. The sensing mechanism relies on the specific catalytic oxidation of glucose by GOX, which generates hydrogen peroxide that etches the AgNPs surface. This etching process attenuates the surface-enhanced Raman scattering (SERS) enhancement effect of AgNPs, resulting in a concentration-dependent decrease in the characteristic SERS signal of GOX at 1343 cm^−1^, allowing quantitative glucose analysis (Figure 8). Furthermore, Wang et al. leveraged the antibacterial properties of silver nanoparticles (AgNPs) to develop a sensor capable of monitoring multiple biomarkers in wound environments. This sensor demonstrated a detection limit of 0.15 mM for glucose. In vivo validation using a murine full-thickness wound model revealed that the AgNP-integrated dressing achieved a 90.35% wound closure rate by day 14. Notably, the system enabled real-time in situ monitoring of dynamic concentration changes of three key wound biomarkers within 3 days [99].

##### Holographic Sensors

**Figure 9:**
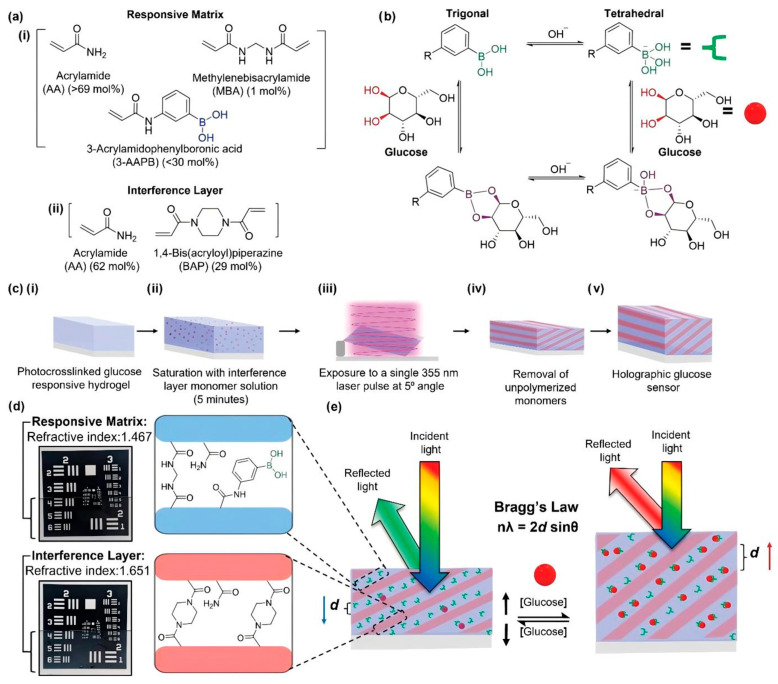
Schematic demonstrating the composition, fabrication, and functional mechanism of HS. (**a**) Chemical structures of each component for the (**i**) RM and the (**ii**) IL; (**b**) reaction pathways for boronic acid binding of glucose in the trigonal and tetrahedral forms; (**c**) schematic highlighting 4 fabrication steps of DP HS, (**i**) photopolymerization of responsive hydrogel, (**ii**) saturation of hydrogel with IL monomer solution and drying, (**iii**) exposure to single 355 nm pulse, and (**iv**) washing of hydrogel to remove unpolymerized material, (**v**) completed holographic glucose sensor; (**d**) hydrogel compositions of RM and IL fringes and their respective RIs, inset photographs depict individual hydrogel covering a section of the USAF resolution target; (**e**) diagram of the working principle of HS upon exposure to glucose, expanding the internal fringe spacing d and changing the reflected wavelength by the Bragg’s law. Reproduced with permission from [100], Advanced Functional Materials, 2023.

Holographic sensors are devices that utilize holographic technology to record diffraction grating structures within hydrogel matrices. These gratings reflect light at specific wavelengths governed by Bragg’s law. The hydrogel incorporates glucose-responsive molecules (e.g., AAPB), where glucose binding induces hydrogel swelling, altering the grating periodicity and consequently shifting the reflected wavelength (color), thereby indicating glucose concentration. Holographic sensors are typically fabricated through two-beam photopolymerization techniques, which record holographic patterns in hydrogel precursor solutions to create diffraction gratings with periodic variations in refractive index or crosslinking density. Reusable by design, sensor performance (e.g., sensitivity, wavelength shift) can be optimized by adjusting hydrogel composition, film thickness, and polymerization conditions. These sensors demonstrate significant potential as components of point-of-care testing devices, offering visual colorimetric readouts [100]. Davies et al. [100] developed a holographic photonic hydrogel sensor (DPHS) composed of a responsive matrix (RM) and interference layer (IL) using acrylamide and 3-AAPB via single-pulse UV-induced two-beam photopolymerization (Figure 9). In this system, the RM containing 3-AAPB enables reversible binding between boronic acid groups and glucose’s cis-diol structure to form cyclic boronate esters. This interaction triggers RM swelling due to charge variations and Donnan osmotic pressure, increasing grating periodicity and inducing redshifted reflection wavelengths according to Bragg’s law for quantitative glucose detection. The IL’s high crosslinking density ensures refractive index contrast and structural stability, while photopatterning enables qualitative identification. This sensor exhibits high sensitivity, low detection limits within physiological concentration ranges, reusability, and strong anti-interference capabilities in urine samples, providing an effective solution for non-invasive, convenient, and cost-effective diabetes monitoring.

Overall, hydrogel-based optical sensors have achieved safe, visualizable, and highly sensitive detection by integrating diverse optical principles (including structural color, fluorescence, surface-enhanced Raman scattering [SERS], localized surface plasmon resonance [LSPR], and holography, as detailed in Table 2) while leveraging their inherent advantages such as flexibility and biocompatibility. This multidisciplinary approach provides a promising direction for advancing next-generation glucose monitoring technologies.

#### 4.1.2. Electrochemical Sensors

Electrochemical sensors, on the other hand, are suitable for real-time monitoring of blood glucose due to their high sensitivity. These sensors detect glucose concentrations by measuring variations in current, voltage, or impedance resulting from electrochemical reactions. Based on the involvement of enzymes in catalytic processes, they are primarily categorized into enzyme-mediated and non-enzyme-mediated systems. Hydrogels play multifunctional roles in electrochemical sensors, including immobilizing enzymes or catalysts, providing a suitable microenvironment, regulating mass transport, enhancing conductivity, and serving as structural scaffolds for integration. Notably, when fabricated into microneedle arrays, hydrogels can achieve minimally invasive sampling capabilities [101,102].

##### Enzyme-Catalyzed Electrochemical Glucose Sensors

**Figure 10:**
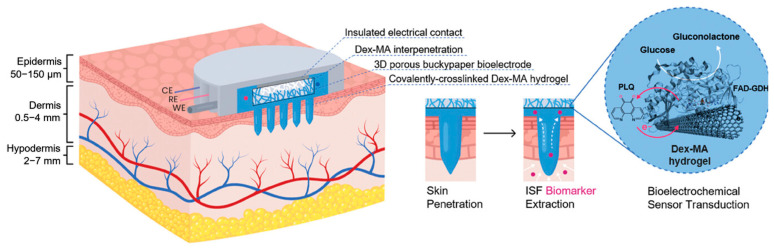
Transdermal monitoring principle of dextran-based hydrogel microneedle array with interpenetrating bioelectroenzymatic sensor. Dex-MA hydrogel microneedles penetrate the epidermis to reach the dermis, extracting interstitial fluid (ISF) through osmosis. The 3D porous buckypaper bioelectrode embedded within the microneedles is loaded with FAD-GDH and PLQ. FAD-GDH catalyzes the oxidation of glucose to gluconolactone, while PLQ, acting as a redox mediator, transfers electrons to the electrode, generating an electrical current signal to achieve electrochemical detection of glucose. The covalently crosslinked Dex-MA hydrogel network interpenetrates with the electrode, not only protecting the electrode from matrix interference but also providing channels for glucose diffusion and electron transfer, thus completing the entire monitoring process from skin penetration, biomarker extraction to signal transduction. Reproduced with permission from [102], Advanced Healthcare Materials, 2024.

In enzyme-catalyzed electrochemical glucose sensors, hydrogels are primarily employed for enzyme immobilization (e.g., GOx or Flavin adenine dinucleotide-dependent glucose dehydrogenase (FAD-GDH)), providing a biocompatible microenvironment and regulating mass transport. For instance, Xu et al. developed a conductive PEDOT:PSS hydrogel immobilized with GOx, integrated with Prussian Blue (PB) as a redox mediator, enabling glucose detection through electrochemical reduction current signal measurement [103]. GOx enzymes are typically incorporated into hydrogels, with sensor performance optimized by adjusting pH and matrix composition. A sophisticated enzymatic catalytic sensor architecture combines reduced graphene oxide (RGO)/silk fibroin (SF) flexible conductive films, Prussian Blue (PB), GOx, and chitosan hydrogels. In this system, the chitosan hydrogel encapsulates GOx to maintain its bioactivity, while the RGO/SF composite film serves as a flexible conductive substrate [104]. Additionally, conductive DNA hydrogel-based sensors integrated with GOx utilize self-assembled conductive networks (e.g., Ag-cytidine-carbon nanotube composites) for enzyme immobilization and electron transfer pathways, with glucose quantification achieved through electrochemical current response analysis [105]. In organic electrochemical transistor (OECT)-based sensors, the GOx hydrogel functions as both a dielectric layer connecting the gate electrode and OECT channel, and as a glucose diffusion pathway. The OECT acts as a biochemical signal amplifier, where glucose binding modulates channel conductivity through current variation [81]. Furthermore, Darmau et al. [102] developed a dextran methacrylate (Dex-MA)-based hydrogel microneedle array integrated with FAD-GDH and phenanthroline quinone (PLQ)-modified carbon nanotube paper biosensors. This system monitors glucose through enzymatic oxidation-induced current changes, with the microneedle array enabling subcutaneous ISF sampling while the hydrogel matrix stabilizes enzymes and redox mediators (Figure 10). Additionally, Yan et al. proposed a glucose-sensing bioelectronic suture based on fiber-based biofuel cells (FiberBFC), where porous gold-plated cotton fibers serve as the foundational electrode material. The fiber anode employs GOx as the catalyst and tetrathiafulvalene (TTF) as the electron mediator for glucose oxidation, enabling real-time in situ monitoring of glucose levels at wound sites [106]. This enzymatic catalytic electrochemical sensor system, when integrated with appropriate biomedical materials, holds promise for the in situ monitoring of diabetic wounds.

##### Non-Enzymatic Catalytic Electrochemical Glucose Sensors

**Figure 11:**
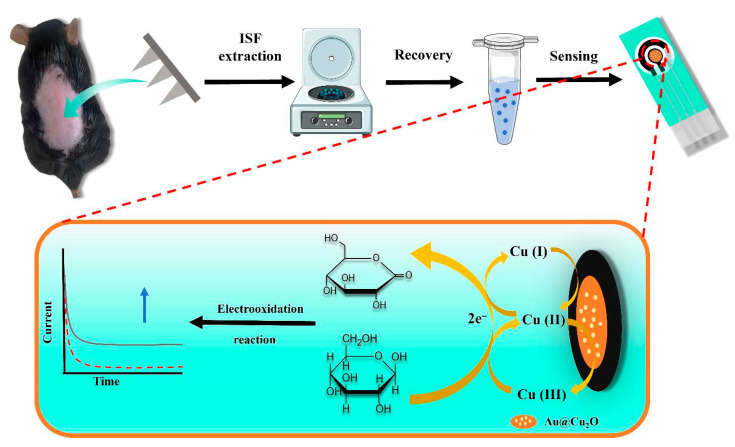
Principle of non-enzymatic electrochemical glucose sensing based on hydrogel microneedle patches and Au/Cu_2_O nanosphere-modified electrodes. MeHA hydrogel microneedles penetrate the skin to extract ISF. Glucose in the ISF undergoes oxidation catalyzed by Au/Cu_2_O nanospheres: Cu^+^ is oxidized to Cu^2+^ and further to Cu^3+^, and Cu^3+^, as a strong oxidizing agent, oxidizes glucose to gluconolactone while being reduced itself, forming a redox cycle and generating current signals. The current varies with glucose concentration, enabling quantitative detection of glucose in ISF by monitoring the current, thus completing the entire process from ISF extraction to signal sensing. Reproduced with permission from [107], Microchemical Journal, 2024.

Compared to enzymatic catalysis, non-enzymatic electrochemical glucose sensors directly utilize catalytic materials (typically noble metals or metal oxide nanomaterials) to catalyze glucose oxidation at the electrode surface, generating current signals. In this context, hydrogels are primarily employed to immobilize catalytic materials and provide stable electrode interfaces. For instance, Jin et al. [107] developed a hydrogel microneedle patch based on methacrylic acid-hyaluronic acid (MeHA) to extract ISF, combined with Au/Cu_2_O nanospheres as electrocatalysts modified on screen-printed carbon electrodes (SPCE). The system achieved non-enzymatic electrochemical detection through redox cycling of Cu^+^/Cu^2+^/Cu^3+^ species, directly catalyzing glucose oxidation to produce current signals (Figure 11). Alternatively, Pt/MXene nanomaterials (Pt nanoparticles loaded on MXene nanosheets) were immobilized in conductive MXene-PVA hydrogels to enhance sensor stability, with a three-electrode system fabricated on a flexible polyimide substrate for detection [82]. Additionally, platinum metal hydrogels (Pt metal hydrogel, PMH) have been attached to the surface of orthodontic appliance-integrated screen-printed carbon electrodes for direct amperometric detection of glucose oxidation currents via chronoamperometry [101]. These non-enzymatic catalytic approaches, independent of enzyme activity, generally exhibit superior stability and durability. However, they may face interference from other electroactive substances, necessitating strategies such as electrode modification layers (e.g., Nafion coatings [82,101,104]) or optimization of electrode materials and working potentials to improve selectivity [102,105,107].

In summary, both hydrogel-based optical sensors and electrochemical sensors leverage the multifunctionality of hydrogels to achieve sensitive glucose detection. Optical sensors offer diverse response modes, ranging from visual color changes to highly sensitive signal outputs. Electrochemical sensors typically demonstrate superior sensitivity and real-time monitoring capabilities, and can be categorized into enzyme-mediated and non-enzyme-mediated types depending on enzymatic involvement. In these systems, hydrogels function not merely as structural scaffolds but also as critical components enabling glucose-specific responsiveness, signal transduction, and efficient biological interfacing.

Despite significant advancements, practical applications still face challenges including long-term in vivo stability, biofouling resistance, calibration requirements, and achieving precise quantitative readouts. Future research directions may focus on further optimizing biocompatibility and stability of hydrogel materials, enhancing sensor sensitivity and specificity, developing more integrated and user-friendly readout devices, and exploring multifunctional integrated sensors (e.g., simultaneous monitoring of glucose, pH, and temperature) for applications in diabetes management and wound healing monitoring [86,90].

### 4.2. pH-Responsive Integrated Smart Monitoring Strategy

Hydrogels not only reflect the pathological status of diabetic wounds through glucose monitoring but also indirectly indicate wound recovery dynamics via pH-responsive mechanisms. By transducing pH fluctuations in the wound microenvironment into detectable signals, these systems enable dynamic pathological evaluation and real-time feedback through colorimetric changes or electrochemical signal transmission, thereby facilitating deeper understanding of wound healing progression and guiding therapeutic interventions [108,109,110]. Their core applications center on diversified integrated strategies for real-time monitoring, pre-warning, and stage-specific assessment.

**Figure 12:**
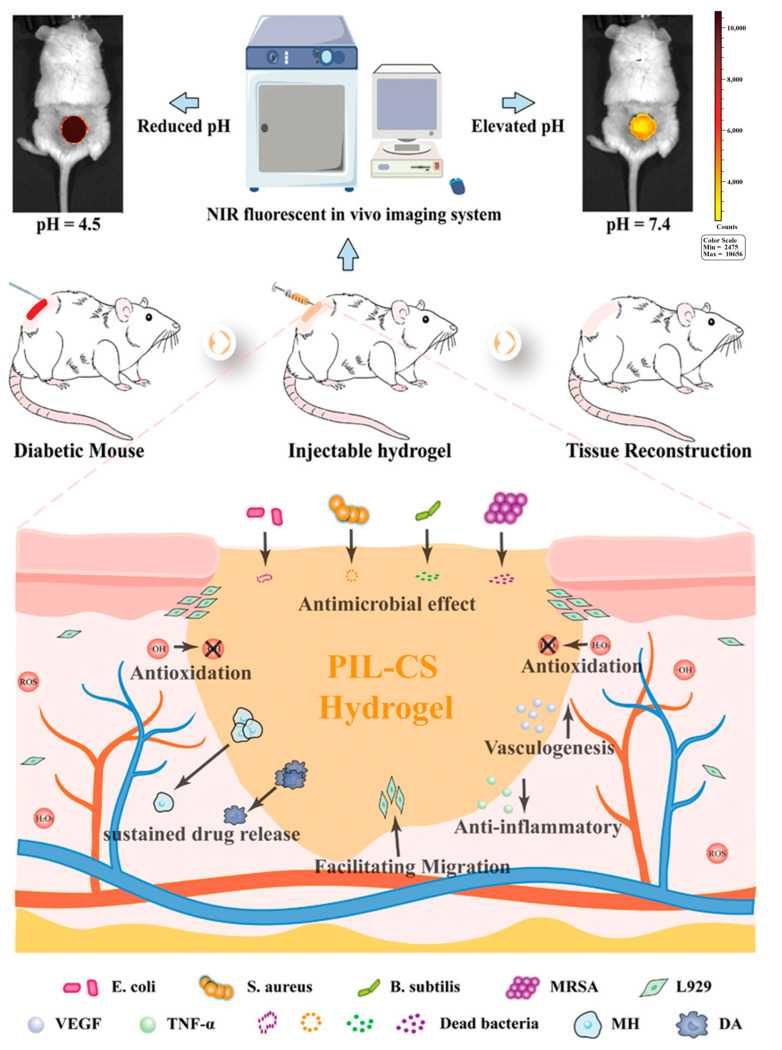
The PIL-CS hydrogel enables real-time monitoring of wound microenvironment pH (4.5–7.4) through CyO fluorescence intensity variations (exhibiting a robust linear correlation with pH) under a near-infrared fluorescence imaging system, while demonstrating multifunctional therapeutic capabilities including effective antibacterial activity, antioxidant properties, sustained drug delivery, enhanced cell migration, anti-inflammatory effects, and angiogenic promotion, thereby synergistically accelerating diabetic wound healing. Reproduced with permission from [111], Advanced Healthcare Materials, 2023.

Notably, real-time point-of-care monitoring represents a critical application domain, where streamlined signal acquisition and analysis meet clinical demands for immediate evaluation. For instance, the GelDerm dressing developed by Mirani et al. utilizes the pH-sensitive properties of natural pigments like red cabbage juice to produce visually perceptible color changes that mirror wound pH conditions. Similarly, poly-carboxybetaine (PCB) hydrogels incorporated with phenol red demonstrate pronounced chromatic transitions within pH 4–8, enabling semi-quantitative analysis through smartphone-captured RGB (Red–Green–Blue) signals for rapid bedside diagnosis [12]. For deeper tissue monitoring, Zong et al. [111] developed a pH-responsive near-infrared (NIR) fluorescent probe CyO-integrated polyionic liquid-chitosan (PIL-CS) hydrogel dressing. This hydrogel architecture comprises a chitosan backbone grafted with dihydrocaffeamic acid (DA) and L-arginine (LAG), alongside a polyionic liquid (PIL-CHO) backbone linked with metformin hydrochloride (MH) through Schiff base structures. Additionally, the NIR fluorescent probe CyO was incorporated into the network via amide bonds. Exploiting the principle of CyO’s fluorescence intensity variation within pH 4.5–7.4, this system synergistically combines real-time imaging technology with smartphones to overcome tissue penetration limitations, enabling highly sensitive, specific, and reversible pH-responsive monitoring of wound microenvironmental changes. This innovation provides real-time monitoring capabilities for diabetic complex wounds while simultaneously demonstrating multifunctional therapeutic properties including antibacterial activity, antioxidant effects, pH-responsive drug release, enhanced cell migration, and angiogenesis promotion (Figure 12).

**Figure 13:**
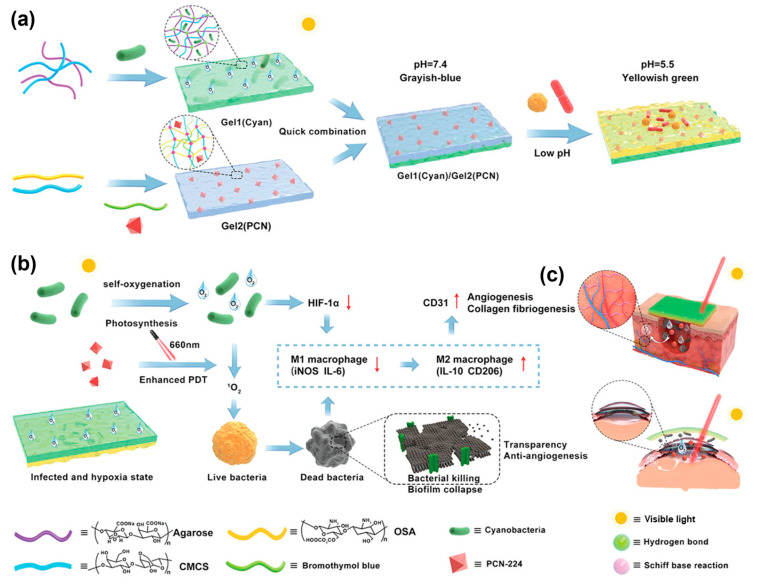
Schematic illustration of the synthesis and mechanism of action of a multifunctional bilayer hydrogel. (**a**) Synthesis schematic of the bilayer hydrogel; (**b**) demonstration of multifunctional properties exhibited by the bilayer hydrogel; (**c**) therapeutic applications of the bilayer hydrogel in diabetic wound healing and refractory keratitis treatment. Reproduced with permission from [116], Advanced Functional Materials, 2022.

In addition, infection risk constitutes one of the core applications of pH-responsive strategies, achieving early intervention through precise detection of pH threshold variations. Healthy wounds typically maintain a weakly acidic environment with pH 4.0–6.5 (normal skin generally ranges at pH 4.0–6.0 or 4.8–5.7), whereas pH levels exceeding 6.5 often indicate infection risks. Chronic wounds (e.g., diabetic foot ulcers) frequently exhibit alkaline conditions (pH 7.0–9.0), which not only facilitate bacterial infections but also disrupt healing processes including inflammatory responses, collagen deposition, and angiogenesis [112,113,114,115]. Based on this principle, anthocyanin-loaded nanofibrous hydrogels display red/violet hues within pH 4–6.5, transitioning to green/cyan when pH exceeds 6.5, thereby providing an intuitive visual signal for infection status [113]. Zhu et al. [116] developed a bilayer hydrogel, where the inner layer is composed of a sodium periodate-oxidized alginate/carboxymethyl chitosan matrix (cross-linked via Schiff base formation), integrated with a light-driven metal–organic framework (PCN-224) and pH indicator (bromothymol blue), while the outer layer consists of agarose and carboxymethyl chitosan (CMCS) loaded with photosynthetic cyanobacterial cells, to respond to infectious microenvironmental changes. The pH indicator visualizes bacterial infection-induced acid–base fluctuations, enabling non-invasive monitoring for timely intervention. When pH decreases from 7.0 to 4.5, the color shifts from grayish blue to pale yellowish green, with this chromatic transition aiding in determining whether the wound microenvironment has entered infection-associated weak acidity, thus providing evidence for early anti-infective treatments (Figure 13).

**Figure 14:**
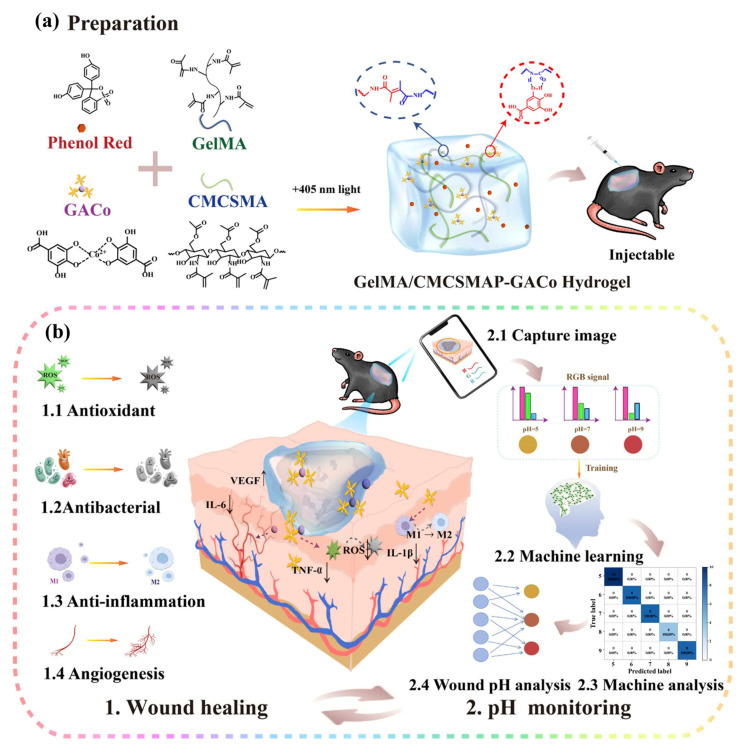
(**a**) Schematic illustration of the synthesis of GelMA/CMCSMAP-GACo hydrogel; (**b**) schematic illustration of the potential application mechanism in diabetic wound healing. Reproduced with permission from [110], Nano Today, 2025.

The dynamic assessment of the healing phase relies on precise monitoring of pH fluctuations, where such integrated strategies enable quantitative analysis of wound healing progression through high-precision signal transduction. For instance, anthocyanin-based hydrogel systems exhibit color transitions within the physiological wound pH range of 4.0–6.5, while visible chromatic shifts synchronously reflect tissue repair dynamics as wounds transition to later healing or recovery stages accompanied by pH normalization. Another pH-responsive system, an asparagine-polylysine point-doped agarose hydrogel array, discriminates between healthy (pH 4.0–6.0) and chronic wounds (pH 7.0–9.0) through pH-dependent optical absorption characteristics, providing quantitative criteria for healing phase stratification [113,117]. Xie et al. developed a self-healing hydrogel (CMC-Eu-EDTA) by crosslinking carboxymethyl cellulose (CMC) with pre-complexed europium-ethylenediaminetetraacetic acid (Eu-EDTA), which integrates enhanced angiogenesis and real-time monitoring capabilities. This hydrogel achieves high-accuracy pH detection through the intensity ratio variation of characteristic emission peaks at 594 nm and 615 nm from the Eu-EDTA complex, with its visible color changes combined with self-healing and injectable properties particularly suitable for dynamic monitoring of complex wound healing phases [118]. Recently, Deng et al. [110] developed an intelligent GelMA/CMCSMA-GACo hydrogel system based on gelatin methacryloyl (GelMA) and chitosan methacrylate (CMCSMA) matrices crosslinked by 405 nm blue light to form a double-network architecture. This system incorporates cobalt-gallic acid metal-phenolic nanoparticles (GACoMPNs) and phenol red as dual functional components. The GACoMPNs exert multiple therapeutic effects through the sustained release of gallic acid and Co^2+^ ions, including antibacterial, anti-inflammatory, and antioxidant properties and angiogenic promotion. The phenol red component serves as a pH-responsive indicator, establishing the hydrogel’s intelligent monitoring core with specific targeting capability. Within the physiological pH range of diabetic wounds (pH 5–9), the hydrogel exhibits distinct color transitions from yellow to orange and finally to red with increasing pH levels, enabling visual tracking of wound microenvironment pH fluctuations and correlation with healing stages. To achieve precise monitoring, the study integrated smartphone-based image acquisition with machine learning algorithms, quantitatively analyzing RGB signal profiles from hydrogel images to assess wound pH with 96% predictive accuracy (Figure 14). Experimental results demonstrated stable colorimetric responses across varying pH wound exudates, enabling long-term dynamic pH tracking during healing processes. This innovation provides reliable real-time indicators for assessing wound healing stages, significantly advancing the intelligence and precision of diabetic wound management [110].

Label-free integrated monitoring represents another critical application scenario, enabling simultaneous sensing of pH and other analytes through structural design. Hydrogels based on photonic crystals or interferometric structures utilize pH-induced network swelling/shrinking effects to modulate the lattice spacing of their internal periodic architectures, thereby achieving monitoring through alterations in the reflected light wavelength (color). This strategy eliminates the need for dye/probe doping while enabling concurrent responsiveness to pH and analytes such as glucose, offering a non-invasive integrated solution for multi-parameter monitoring of wound environments [119].

### 4.3. Multiple Signal Monitoring Strategy

**Figure 15:**
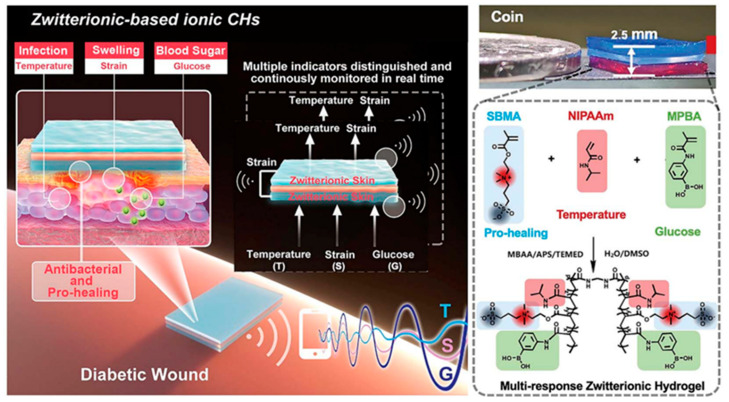
Zwitterion-based conductive hydrogels. Reproduced with permission from [122], Advanced Functional Materials, 2021.

In addition to monitoring common indicators such as glucose and pH levels, parameters including wound temperature, stress, humidity, and exudate are also applicable for diabetic wound surveillance [120]. In this context, Zeng et al. developed a conductive biogel using xanthan gum, squid ink nanoparticles, and silver nanowires. This biogel not only scavenges reactive oxygen species (ROS) to improve the microenvironment of diabetic wounds but also enables real-time monitoring of skin temperature and electromyographic signals at the wound site [121]. Building upon this, Guo et al. constructed a zwitterionic carboxybetaine (SBMA)-based skin sensor system with a sandwiched architecture. The system comprises an upper thermoresponsive poly (N-isopropylacrylamide) (PNIPAM) hydrogel layer, which expels excess water through hydrophobic interactions upon temperature elevation, thereby increasing ion concentration and reducing resistance. The bottom layer incorporates methacrylamide phenylboronic acid (MPBA) for glucose-responsive detection. An insulating intermediate layer ensures decoupling of temperature and glucose signals. Notably, the capacitance variation across the hydrogel dressing reflects mechanical pressure at the wound site. This integrated hydrogel system enables continuous differentiation of temperature, mechanical stress, and glucose information, achieving real-time monitoring of diabetic wound infection, swelling, and glycemic levels (Figure 15) [122]. To expand wound information acquisition, Zheng et al. [117] developed a sensor-array hydrogel matrix. By integrating urea, uric acid, and total protein detection into a carbon-dot-doped hydrogel microfluidic patch, they constructed enzyme sensors (for glucose, urea, and uric acid) and dye sensors (for pH and total protein), enabling high-precision monitoring of five biomarkers. Distinct color patterns from this sensor array clearly differentiate healing and non-healing zones while assessing wound inflammation and infection status.

### 4.4. Integrated Diagnosis and Treatment Strategy

**Figure 16:**
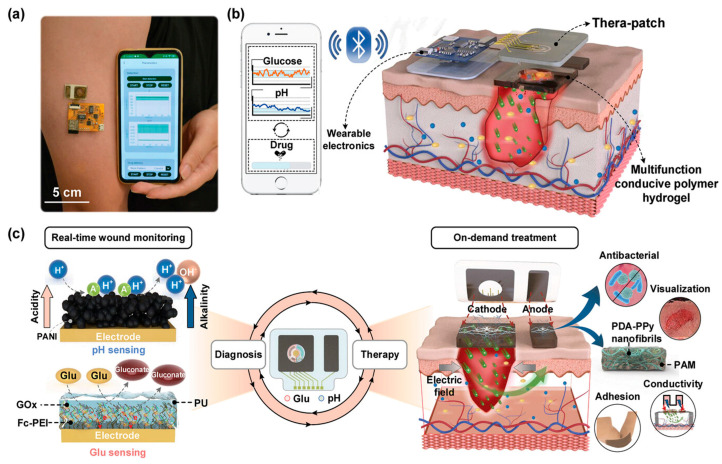
(**a**) Photograph of the fully integrated wireless diabetic wound theranostic system; (**b**) schematic illustration of wireless theranostic wound management system, including a customized smartphone App, a wearable electronic device, and a Thera-patch. The Thera-patch continuously monitors the wound state and executes on-demand treatment via combining iontophoresis with MFCPH. The wearable electronic device collects the sensing data and receives App commands to trigger drug delivery. Smartphone App receives the sensing data, analyzes the diabetic wound state, and sends drug delivery command; (**c**) The closed-loop wound theranostic administration strategy of the management system for real-time wound monitoring and on-demand treatment. Reproduced with permission from [125], Advanced Functional Materials, 2024.

With the advancement of integrated diagnostic-therapeutic technologies, numerous smart gel-based dressings have been developed to autonomously monitor temperature and humidity levels in wound exudates. Based on these sensing results, patients or clinicians can operate smartphones to trigger on-demand drug release [123]. Furthermore, researchers innovatively designed a dual-layer “test-and-treat” pad for monitoring drug-resistant/sensitive (DR/DS) bacterial infections in diabetic wounds and delivering differentiated therapies. The inner layer of this hydrogel integrates bromothymol blue (a pH infection indicator), nitrocefin (a sβ-lactamase indicator), and acid-responsive drugs (Fe-carboxypenicillin framework). The outer layer combines mechanoluminescent materials (ML, CaZnOS:Mn^2+^) with visible-light-responsive photocatalysts (Pt@TiO_2_) embedded in elastic polydimethylsiloxane. According to colorimetric sensing outcomes, acid-responsive drugs enable chemical eradication of DS bacteria, while DR bacterial infections can be addressed through mechanoluminescence-activated photodynamic therapy (PDT) via Pt@TiO_2_ under manual pressure [124]. More intriguingly, recent studies have reported a wireless theranostic wound management system for closed-loop diabetic wound care [125]. This system comprises a customized smartphone application, wearable electronic devices, and multifunctional hydrogels (Figure 16a,b), enabling real-time and simultaneous detection of wound pH and glucose levels. The collected pH and glucose data are processed by the wearable device and transmitted to the smartphone app via Bluetooth. The app analyzes the chronic wound status based on sensor data. When glucose levels exceed the threshold, the app sends activation commands to the electronic device, triggering insulin delivery through iontophoresis. This closed-loop process effectively reduces glucose levels and promotes wound healing (Figure 16c).

## 5. Conclusions and Prospects

### 5.1. Conclusions

In summary, as a critical component in the treatment of diabetic wounds, hydrogels not only immobilize active materials but also confer flexibility, biocompatibility, and functionalities such as auxiliary sampling and monitoring to sensors, making them an optimal solution for enhancing sensor performance and expanding healthcare applications. Based on the unique physiological characteristics of diabetic wounds, this review systematically discusses preparation methods and working principles of various smart hydrogels, critically analyzes their evolutionary trajectory from intelligent response to smart monitoring, followed by multifunctional integration and realization of integrated diagnosis and treatment. Challenges related to sensitivity, selectivity, stability, wearable comfort, and minimally invasive properties are also evaluated. By exploring innovative advancements in systematization, miniaturization, and integration of hydrogels, this work highlights strategies to continuously enhance their capacity for wound sensing and adaptation, thereby providing personalized solutions for the diagnosis and treatment of chronic wounds such as diabetic foot ulcers.

### 5.2. Challenges

Hydrogel-based biosensors demonstrate promising prospects in diabetic wound management, yet face significant challenges including long-term in vivo stability and biofouling. Hydrogels, requiring sustained functionality in complex biological environments, are prone to bacterial colonization and dehydration during prolonged use, which compromises their operational lifespan. Moreover, biofilm formation on sensor surfaces can degrade analytical accuracy. For precise calibration and quantitative readouts, particularly in colorimetric sensors, achieving reliable quantitative measurements remains a critical technical hurdle. The inactivation of GOx and the generation of inflammatory byproducts such as H_2_O_2_ further necessitate resolution.

Current non-invasive glucose monitoring technologies are susceptible to multiple interfering factors, while the correlation between interstitial fluid glucose levels and actual blood glucose concentrations remains poor (e.g., sweat glucose concentrations may differ from blood levels by up to 100-fold). Therefore, there is an urgent need for devices with enhanced precision, robust anti-interference capabilities, and user-friendly readout mechanisms. Conventional hydrogels often exhibit insufficient mechanical strength to conform to irregular wound geometries. For drug delivery applications, balancing glucose responsiveness with mechanical durability represents a key design consideration. Additionally, potential risks of nanomaterial-induced drug resistance and the cytotoxicity associated with lectin-based binding agents like concanavalin A require thorough evaluation.

### 5.3. Prospects

In the future, through further optimization of material biocompatibility and stability, this research will focus on developing hydrogel materials with enhanced safety, durability, self-healing capabilities, and stimuli-responsive degradation/release mechanisms (e.g., based on dynamic covalent bonds) to meet the demands of long-term applications. Concurrently, efforts will be directed toward improving monitoring capabilities by exploring novel mechanisms for precise, rapid, and real-time detection of multiple parameters, including glucose, pH, temperature, ROS, and enzyme activity. Advanced sensor technologies, such as photonic crystal-based, fluorescence, and electrochemical systems with high sensitivity and specificity, will be developed. These sensors will integrate multiple responsive strategies to effectively address the complexities of wound environments.

In terms of the development of integrated diagnostic and therapeutic smart dressings, by integrating monitoring and therapeutic functionalities, this innovation aims to achieve glucose regulation through multifaceted synergistic effects including on-demand insulin release, broad-spectrum antibacterial activity, antioxidant and anti-inflammatory actions, angiogenic promotion, and tissue regeneration enhancement. Concurrently, the physical properties of hydrogels will be optimized to improve self-healing capacity, injectability, optical transparency, adhesiveness, and hemostatic performance, thereby enabling adaptation to irregular wounds while minimizing secondary trauma. It is anticipated that deep integration of sensor data with artificial intelligence (AI), big data analytics, and Internet of Things (IoT) technologies could enable personalized diabetes management protocols, potentially advancing diagnostic precision, preventive healthcare, and cost-effective medical solutions.

The continuous exploration of complex mechanisms underlying diabetic wound healing remains pivotal, particularly the specific action mechanisms of multifunctional hydrogels at distinct healing stages (e.g., promoting macrophage M2 polarization) which warrant further elucidation. By addressing these challenges and advancing research in these critical areas, hydrogel-based biosensors hold significant promise to deliver more effective and intelligent solutions for comprehensive management and efficient healing of chronic diabetic wounds.

## Figures and Tables

**Table 1:** Comparative analysis of response mechanisms.

Response Type	Response Mechanism	Composition	Application	Advantage	Ref.
pH Response	1. Solvation or contraction of ionizable groups (-COOH, -NH_2_) of polymers; 2. Acid-sensitive dynamic covalent bond degradation	Alginate, chitosan, carboxymethyl cellulose, oxidized dextran, bovine serum albumin (BSA)	pH-responsive degradation of ODex/BSA-Zn hydrogels via Schiff base bonding	Dynamically adapts to the acidic environment of the wound for precise and targeted drug delivery; some hydrogels can actively regulate pH to promote macrophage polarization	[36,37,38,39]
enzyme Response	Use of protease-sensitive peptide chains, such as MMP-9, as cross-linking units to trigger hydrogel degradation or drug release after enzymatic digestion	Oxidized dextran, carboxymethyl chitosan, MMP-9-sensitive peptide chain	MMP-9 releases M2-type macrophage exosomes in response to hydrogels and promotes M1→M2 polarization to accelerate healing	Specific targeting of inflammatory sites, regulating abnormal enzyme activity, and promoting wound repair	[40]
ROS Response	ROS-sensitive chemical bond (thioketone, borate) breakage triggers degradation or drug release	Tannic acid (TA), polyvinyl alcohol (PVA), cerium dioxide (CeO_2_) nanoenzymes, phenylborate crosslinker	1. HAP-PVA/Reg3α hydrogel degrades and releases the drug in a high hydrogen peroxide (H_2_O_2_) environment; 2. PPBA-TA-PVA hydrogel cascade scavenges ROS	Dual function: responsive drug release + direct removal of oxidative stress to improve the wound microenvironment	[41,42,43,44]
Glucose Response	1. Glucose oxidase (GOx) catalyzes the production of gluconic acid and H_2_O_2_ from glucose; 2. Concanavalin A (ConA) binds competitively to glucose; 3. Phenylboronic acid (PBA) reversibly binds to glucose	GOx, ConA, PBA	1. GOx hydrogel triggers insulin release via pH/ROS changes; 2. PBA hydrogel binds glucose to regulate solubility	Real-time monitoring of blood glucose levels, on-demand drug release, can be integrated with other response mechanisms to build a multi-functional system	[45,46,47]

**Table 2:** Summary of the characteristics of the different types of optical sensors.

Sensor Type	Principle	Composition	Preparation Techniques	Advantage	Limitations	Refs.
PC	Structural color changes due to Bragg diffraction	Hydrogel + PBA/AFPBA + nanostructures	Nano self-assembly, template imprinting, in situ polymerization	Visual color change, reusable, microneedle integration	Slow response (minutes), visible light observation required	[90,91]
Luminous	Change in fluorophore signal (intensity/wavelength)	Hydrogel + fluorophore (NDs/QDs etc.) + enzyme/aptamer/PBA	Hydrogel polymerization of doped fluorophores, enzyme fixation	Continuous monitoring, biocompatibility, high specificity, self-healing capability	An external light source is required for excitation, and long-term stability needs to be improved	[87]
SERS	Precious metal nanoparticles enhance Raman scattering signals	Hydrogel + Ag/Au nanoparticles	Nanoparticle integration into hydrogel microspheres/substrates	Ultra-high sensitivity, possible pre-process-free detection	High cost and signal stability are affected by the environment	[95,98]
LSPR	Localized surface plasmon resonance effects on noble metal nanoparticles	Hydrogel + PBA + Au nanoparticles	Covalent immobilization of nanoparticles, fibre tip polymerization	Fast response (seconds), quantitative monitoring, flexible, high sensitivity	Dependent on nanoparticle uniformity, it requires precision optical inspection equipment	[86]
Holographic	Holographic grating period change induces reflection wavelength change	Hydrogel + PBA + dual photopolymerization layer	Preparation of periodic hydrogel films by two-photopolymerization	Reusable, adjustable sensitivity, instant visualization of readings	Temperature-dependent response time, complex preparation process	[100]

## Data Availability

No new data were created or analyzed in this study. Data sharing is not applicable to this article.

## Abbreviations

3-APBA/3-AAPB	3-acrylamidophenylboronic acid
AA	Acrylic acid
AFPBA	(4-((2-acrylamidoethyl)carbamoyl)-3-fluorophenyl)boronic acid
AGEs	Advanced glycation end-products
AgNPs	Silver nanoparticles
AM	Acrylamide
BSA	Bovine albumin
CD	Carbon dots
CDT	Chemodynamic therapy
CGM	Continuous Glucose Monitoring
CMC-Eu-EDTA	Carboxymethyl cellulose ethylenediaminetetraacetic acid
Con A	Concanavalin A
Dex-MA	Dextran methacrylate
ECM	Extracellular matrix
FAD-GDH	Flavin adenine dinucleotide-dependent glucose dehydrogenase
FRET	Fluorescence Resonance Energy Transfer
GOx	Glucose Oxidase
GSH-Px	Glutathione peroxidase
HAP	Hyaluronic acid
IL	Interference layer
ISF	Interstitial fluid
LSPR	Localized Surface Plasmon Resonance
MeHA	Methacrylated Hyaluronic Acid
MMP-9	Matrix metallopeptidase 9
NDs	Nanodiamonds
NIR	Near Infrared
ODex	Oxidized dextran
OECT	Organic electrochemical transistors
PBA	Phenylboronic acid
PC	Photonic Crystal
PCHs	Photonic crystal hydrogels
PEGDA	Poly(ethylene glycol) diacrylate
PET	Photoinduced Electron Transfer
PLQ	phenanthroline quinone
PMH	Pt metal hydrogel
PVA	Polyvinyl alcohol
QDs	Quantum Dots
Reg3α	Regenerating family protein 3α
RGO	Reduced Graphene Oxide
RM	Responsive matrix
ROS	Reactive oxygen species
SERS	Surface-Enhanced Raman Scattering
SF	Silk Fibroin
SOD	Superoxide dismutase
SPCE	Screen-printed carbon electrode
TA	Tannic acid
